# Simultaneous radiomethylation of [^11^C]harmine and [^11^C]DASB and kinetic modeling approach for serotonergic brain imaging in the same individual

**DOI:** 10.1038/s41598-022-06906-0

**Published:** 2022-02-28

**Authors:** Chrysoula Vraka, Matej Murgaš, Lucas Rischka, Barbara Katharina Geist, Rupert Lanzenberger, Gregor Gryglewski, Thomas Zenz, Wolfgang Wadsak, Markus Mitterhauser, Marcus Hacker, Cécile Philippe, Verena Pichler

**Affiliations:** 1 Division of Nuclear Medicine, Department of Biomedical Imaging and Image-Guided Therapy, Medical University of Vienna, Vienna, Austria; 2 Department of Psychiatry and Psychotherapy, Medical University of Vienna, Vienna, Austria; 3 CBmed GmbH, Center for Biomarker Research in Medicine, Graz, Austria; 4 Ludwig Boltzmann Institute Applied Diagnostics, Vienna, Austria; 5 Department of Pharmaceutical Sciences, Division of Pharmaceutical Chemistry, University of Vienna, Vienna, Austria

**Keywords:** Nuclear chemistry, Chemical synthesis, Neuroscience

## Abstract

Simultaneous characterization of pathologies by multi-tracer positron emission tomography (PET) is among the most promising applications in nuclear medicine. Aim of this work was the simultaneous production of two PET-tracers in one module and test the relevance for human application. [^11^C]harmine and [^11^C]DASB were concurrently synthesized in a ‘two-in-one-pot’ reaction in quality for application. Dual-tracer protocol was simulated using 16 single PET scans in different orders of tracer application separated by different time intervals. Volume of distribution was calculated for single- and dual-tracer measurements using Logan’s plot and arterial input function in 13 brain regions. The ‘two-in-one-pot’ reaction yielded equivalent amounts of both radiotracers with comparable molar activities. The simulations of the dual-tracer application were comparable to the single bolus injections in 13 brain regions, when [^11^C]harmine was applied first and [^11^C]DASB second, with an injection time interval of 45 min (r_xy_ = 0.90). Our study shows the successful simultaneous dual-tracer production leading to decreased radiation burden and costs. The simulation of dual subject injection to quantify the monoamine oxidase-A and serotonin transporter distribution proved its high potential. Multi-tracer imaging may drive more sophisticated study designs and diminish the day-to-day differences in the same individual as well as increase PET scanner efficiency.

## Introduction

Positron emission tomography (PET) often coupled to anatomical imaging modalities such as magnetic resonance imaging (MRI) or computed tomography (CT) is the primary method for functional imaging on a molecular level. Since one PET-tracer delivers limited information, multi-tracer concepts are coming more and more into the spotlight to overcome limitations regarding e.g. tumour heterogeneity^1^. Importantly, rapid multi-tracer applications allow the simultaneous characterization of pharmacokinetics and functional information and at the same time improve cost and time aspects during hospital routine. Hence, a plethora of multi-tracer concepts has been published during the last years focusing on tumour biology^2^. Hitherto, a multi-tracer application has been described for tumour imaging using mostly ^18^F-labelled tracers displaying metabolism, hypoxia, cellular proliferation and tumour blood flow in different combinations. The elegance of a multi-tracer application was also recognized in the research field of psychiatric disorders, where a population-based characterization of multi-tracer brain maps has been published^3^.

While unique energies of different radionuclides provide clearly distinguishable signals in single photon emission tomography (SPECT), coincident photons (all 511 keV) detected as a consequence of annihilation events of all positron emitting radionuclides pose a challenge to assign the signals to different PET tracers applied simultaneously. Therefore, multi-tracer PET imaging requires computational post-processing to recover the single signals from a summed PET image. Current approaches are using mainly PET-nuclides with different half-life or kinetic modelling^2,4,5^.

Usually, PET-tracers for brain imaging are small molecules labelled with short-lived positron emitters, i.e. carbon-11 and fluorine-18 (20 min and 110 min half-life, respectively). Authentic carbon-11 labelling is of special interest, since ‘native’ endogenous ligands (unmodified) are more likely to penetrate the blood brain barrier, the chemical structure is not altered and the radiation burden for the patient is low^6^. Hitherto, multi-tracer compartment modelling was utilized for neuroimaging studies, using two different ^11^C-labelled tracers, either with two rapidly reversible tracers or with one irreversible and one reversible tracer^7–9^. The authors concluded that dual-tracer imaging of two carbon-11 tracers is feasible using continuous dynamic scans with an injection delay of 10–20 min between the tracers^2^. Alternatively, others have recovered individual tracer signals from dual-tracer applications using signal-separation algorithms, extrapolation, simultaneous fitting or a template method^5,8^.

From a logistical point of view, multi-tracer PET studies rely on providing multiple tracers on time, which can be limited by the capability of the production site. Particularly, the number of examinations with ^11^C-labeled tracers is drastically restricted, as only one tracer per synthesizer can be produced within a time frame of approximately 2 h. Cassette-based fully automated synthesis modules with a cassette changing unit are excluded from this calculation, but are rarely applied for carbon-11. According to radiation protection, a minimum decay time of six half-lives (around 2 h) between two carbon-11 syntheses within the same hot-cell is required for safe preparatory work for a second consecutive radiosynthesis. Therefore, in clinical routine and studies during an e.g. eight hours’ workday, only two syntheses of carbon-11 labelled compounds can be safely performed per synthesizer/hot-cell. Furthermore, PET examinations are expensive, e.g. due to high synthesis costs of individual productions for one or two patients (especially for carbon-11 tracers). Consequently, the level of performance of each production site depends and intertwine on the number of productions, personnel, hot cells and synthesizers. Highly optimized radiosyntheses are therefore an economical and logistical factor, and publications of improved protocols are manifold. For instance, a simultaneous synthesis of two different carbon-11 tracers ([^11^C]DASB and [^11^C]methionine) was published by Lee et al.^10^ describing [^11^C]CH_3_I production in one cyclotron run, further split to two different loops loaded with the respective precursor. Such simultaneous, but separated, radiochemical reactions have also been reported for other radiotracers^11,12^. To the best of our knowledge, the introduction of [^11^C]CH_3_I in a solution containing two precursors has not been described so far.

Intermolecular competing reactions, like the ^11^C-methylation of two different precursors within the same solution, are dependent on a set of reaction parameters, like the reactivity of the functional group or kinetic considerations, among others^12^. Such reactions are complex, and it is difficult to predict which product will form preferentially and in which amounts. Even different isotopes show differences in the turnover rate within certain reactions, exploited for example in isotope fractionation^13^. The concept of multi-substrate reaction was applied in basic research for multi-substrate screening in order to identify substrate specific catalysts^14,15^. Our group accomplished for the first time to apply the dual-substrate reaction for highly applied and clinically relevant purposes and in a way, that both products are synthesized in high quality pharmaceutical grade for intravenous application.

Most ^11^C-methylations using [^11^C]CH_3_I or [^11^C]CH_3_OTf take place via a nucleophilic substitution reaction. For nucleophilic substitution reactions there are four reaction parameters defining the outcome of the reaction: the nucleophilic character of the nucleophile, the substrate, the quality of the leaving group and the solvent. This applies also for the substoichiometrical radiosynthesis of the PET-tracers described herein.

**Figure 1:**
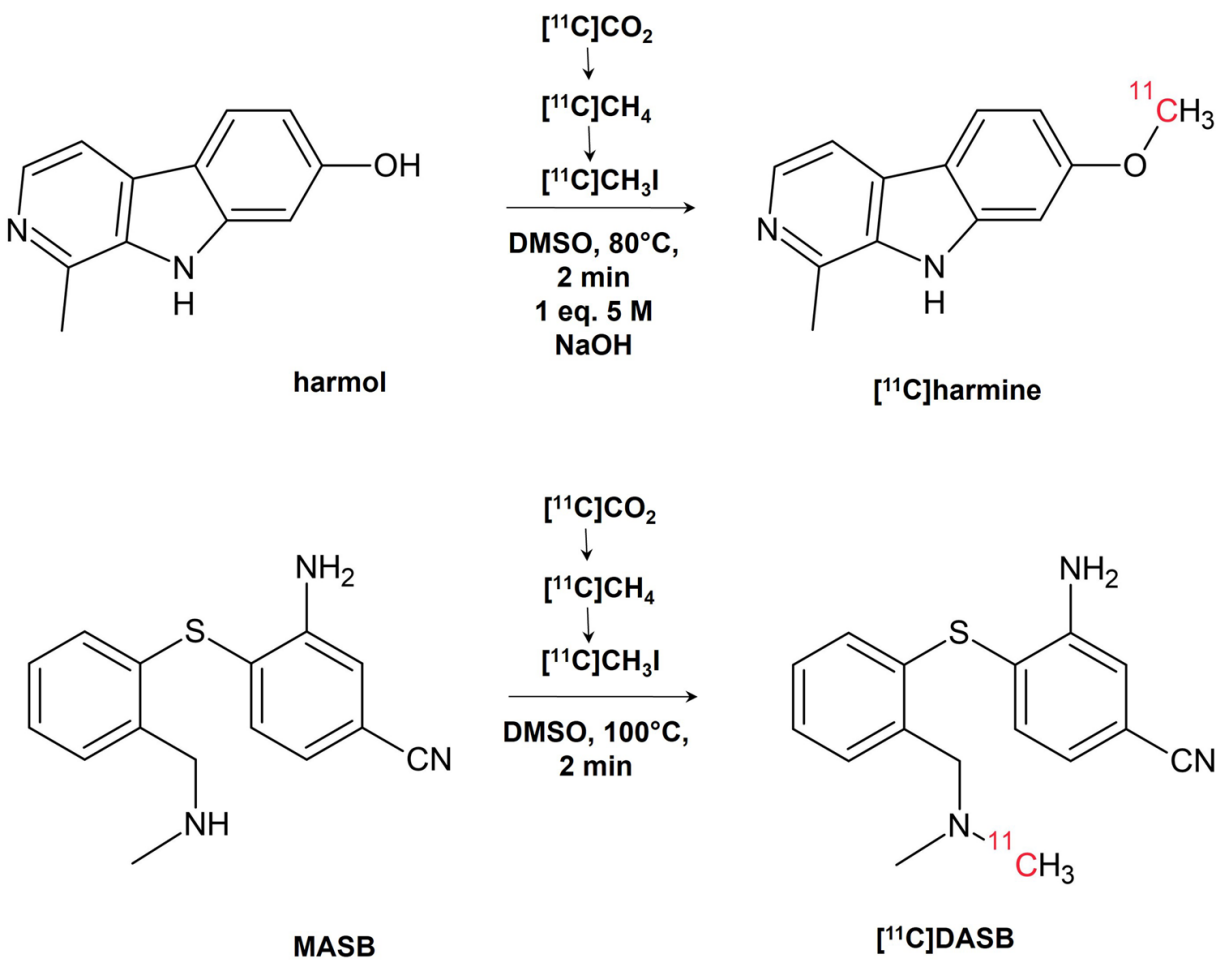
Reaction conditions of the PET brain tracers [^11^C]harmine and [^11^C]DASB.

For the conversion of the respective precursors to the products [^11^C]harmine and [^11^C]DASB, respectively, [^11^C]CH_3_I in dimethyl sulfoxide (DMSO) is used as previously described and illustrated in Fig. 1^16,17^. The possible difference in product formation is primarily dependent on the nucleophile (phenol vs. alkyl amine) as used solvent (DMSO), temperature (100 °C), leaving group (iodide) and substrate ([^11^C]CH_3_I) are constant in a one-pot synthesis.

Both radiotracers are used in clinical investigations in patients suffering from major depression as they identify pathophysiological changes associated with the serotonergic system. More precisely, [^11^C]harmine binds to the enzyme monoamine oxidase A (MAO-A), which is responsible for serotonin degradation, and therefore a measure for MAO-A distribution. [^11^C]DASB binds to serotonin transporters (SERT) and could be used for their quantification on protein level. Studying two or more biological processes requires multiple scans including the setback of several productions and increased radiation burden for personnel, patient and synthesizer, if PET/CT is used. Dual-tracer application has the potential to overcome these difficulties, while obtaining more than one functional information of the same subject and at the same time point (identical physiological state). Previous studies showed that [^11^C]harmine and [^11^C]DASB have not only intra-individual variations, but also high seasonal variations of the binding potential in the same subject^18–20^. Indeed, imaging studies using longitudinal measurements (with interventions) and validation of treatment response or disease progress are hardly executable. To this end, simultaneous measurement of two targets would allow for complex study designs while leading to an efficiency enhancement of PET-scanner capacity.

**Figure 2:**
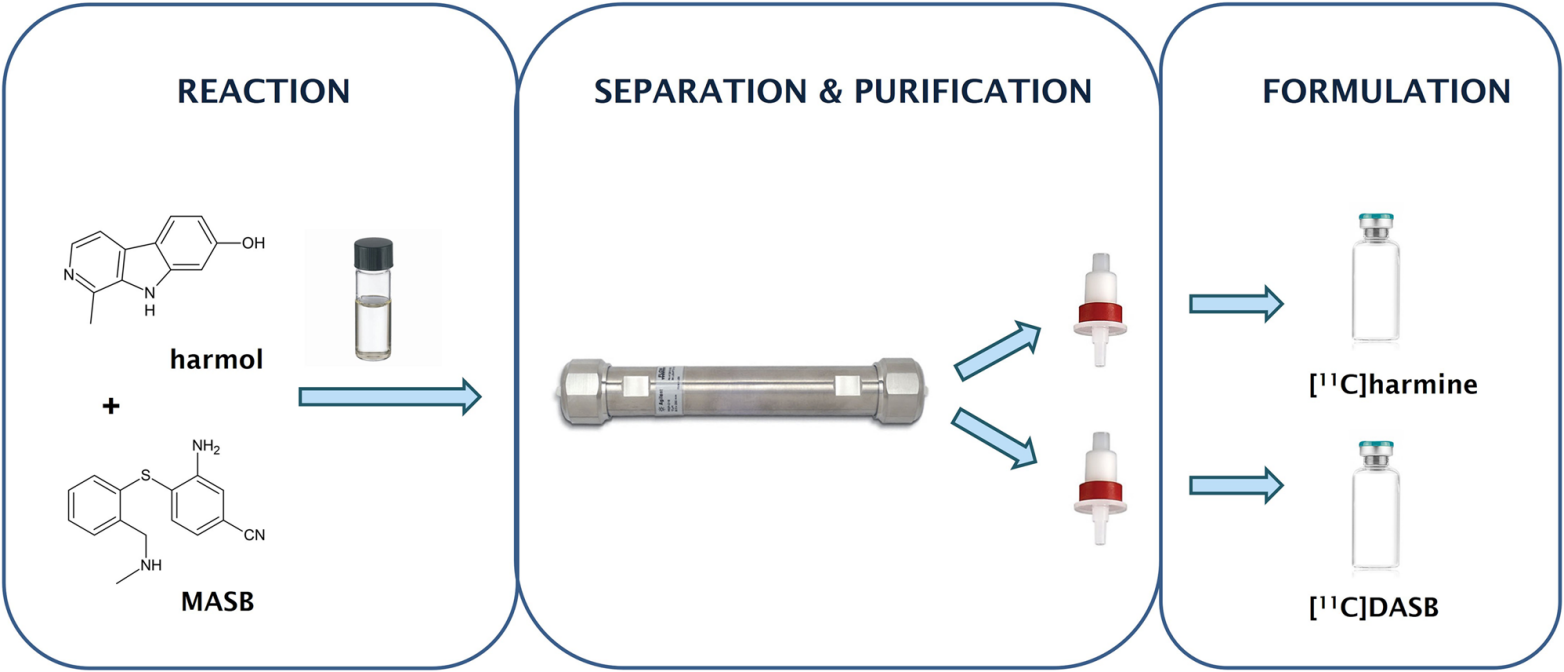
Illustration of the ‘two-in-one-pot’-concept. Two reactions are performed in the same reaction vial, using two different precursors and the same reactant (e.g. [^11^C]CH_3_I). In the second step, the separation takes place on a semi-preparative HPLC column, the pure products are purified from the mobile phase and further formulated to separate solutions, which can be applied to patients.

The aim of this study was to improve and accelerate clinical investigations in patients suffering from major depression and other neuropsychiatric diseases with altered serotonergic neurotransmission by the simultaneous production of two ^11^C-labled PET-tracers using a ‘two-in-one-pot’ method for the first time (Fig. 2). Consequently, reaction time, cost and radiation burden for the producer shall be reduced. Additionally, we hypothesize that [^11^C]harmine and [^11^C]DASB can be used for dual-tracer application.

## Results

### Radiochemistry

All radiopharmaceutical terms are used according to the published consensus^21^. Both PET-tracers were prepared simultaneously in a ‘two-in-one-pot’ reaction and successfully purified using one single RP-HPLC run. Radiochemical yields @EOB were 2.0 ± 0.3 GBq (2.3 ± 0.5% not corrected for decay; based on [^11^C]CO_2_) for [^11^C]DASB and 2.0 ± 0.7 GBq (2.2 ± 0.8%) for [^11^C]harmine, respectively. Hence, both products were obtained in the same amount. Compared to single synthesis results (Table 1) overall radiochemical yield is in the same range. The yield of each tracer was sufficient for application in 1–2 patients or for DUAL application in one patient.

**Table 1:** Comparison of the results obtained for DUAL and single radiotracer production.

	Dual synthesis		Single synthesis	
	[^11^C]harmine (n = 3)	[^11^C]DASB (n = 3)	[^11^C]harmine (n = 42)	[^11^C]DASB (n = 73)
Activity yield (GBq)	2.0 ± 0.7	2.0 ± 0.3	4.8 ± 2.1	5.1 ± 1.7
RCY based on [^11^C]CO_2_@EOB	2.2 ± 0.8%	2.2 ± 0.5%	4.1 ± 1.8%	4.1 ± 1.2%
Radiochemical purity	> 99%	> 97%	> 99%	> 97%
Molar activity (GBq/µmol)	103.6 ± 63.1	178.3 ± 17.9	132.2 ± 85.6	98.5 ± 72.4
Residual precursor (µg/mL)	0.19 ± 0.05	0.11 ± 0.10	0.11 ± 0.10	0.29 ± 0.76

### Quality control

All productions of the dual synthesized products [^11^C]harmine and [^11^C]DASB were of comparable quality to conventional production. Additionally, the molar activity and the residual precursor concentration were in the same range as resulted in single batch productions (see Table 1). A new RP-HPLC assay was established to separate precursors (harmol 0.39 min and MASB 1.35 min) and products (harmine 2.14 min and DASB 3.36 min) within a single 5-min run, in contrast to the two methods used for the single batch production with 3 min runtime each. Cross-contamination of products was not observed for all production batches, neither in the single run quality control (QC) assays nor in the optimized RP-HPLC assay (QC-DUAL) (Suppl. Table 1). Method validation features comparable performance of the QC-DUAL and the single-HPLC assays.

### Simulation of DUAL-tracer application

**Figure 3:**
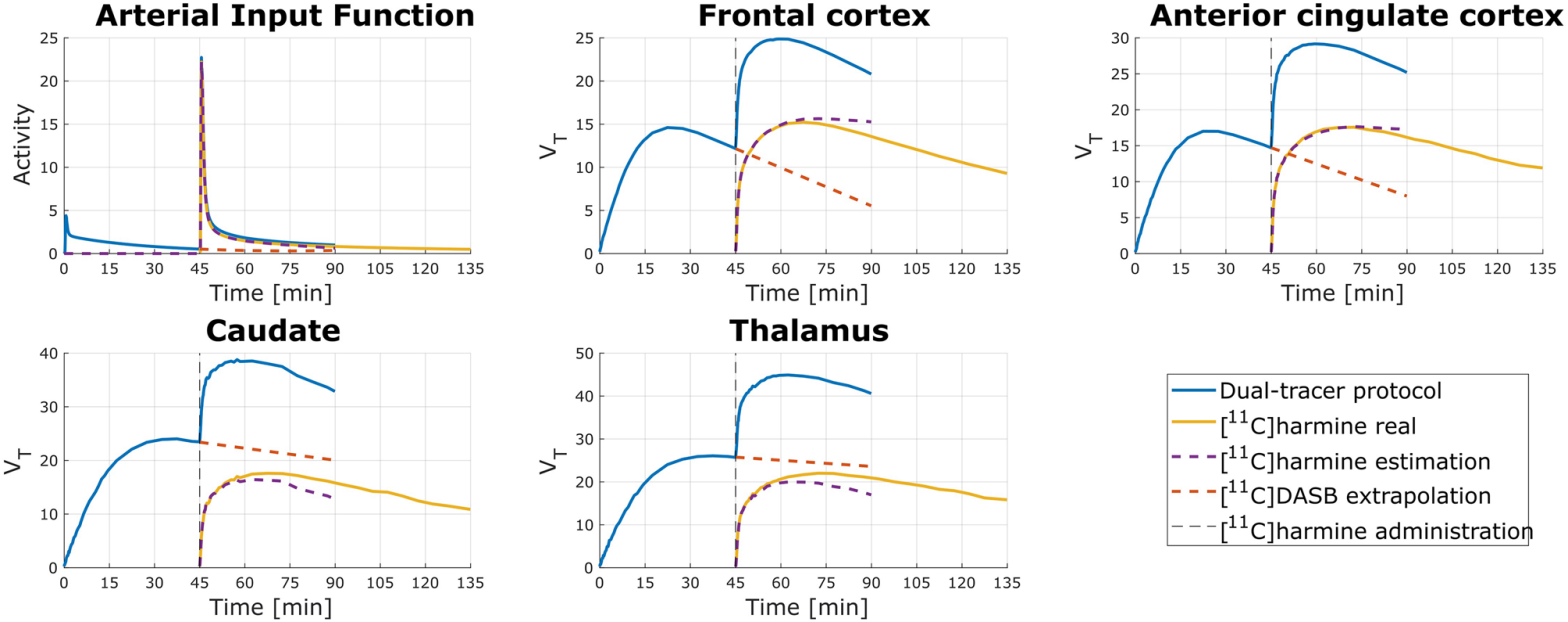
Example TACs and AIFs (top left) when [^11^C]DASB was applied as first. Blue lines are representing dual-tracer application (TAC_DASB_HAR_ and AIF_DASB_HAR_), yellow lines are showing TAC_HAR_ and AIF_HAR_ of 90 min [^11^C]harmine measurement, purple dashed lines shows a time course of estimated TAC_HAR_est_ and AIF_HAR_est_ and the red dashed line express extrapolated TAC_DASB_ext_ and AIF_DASB_ext_. A black vertical dashed line denotes the administration of [^11^C]harmine.

**Figure 4:**
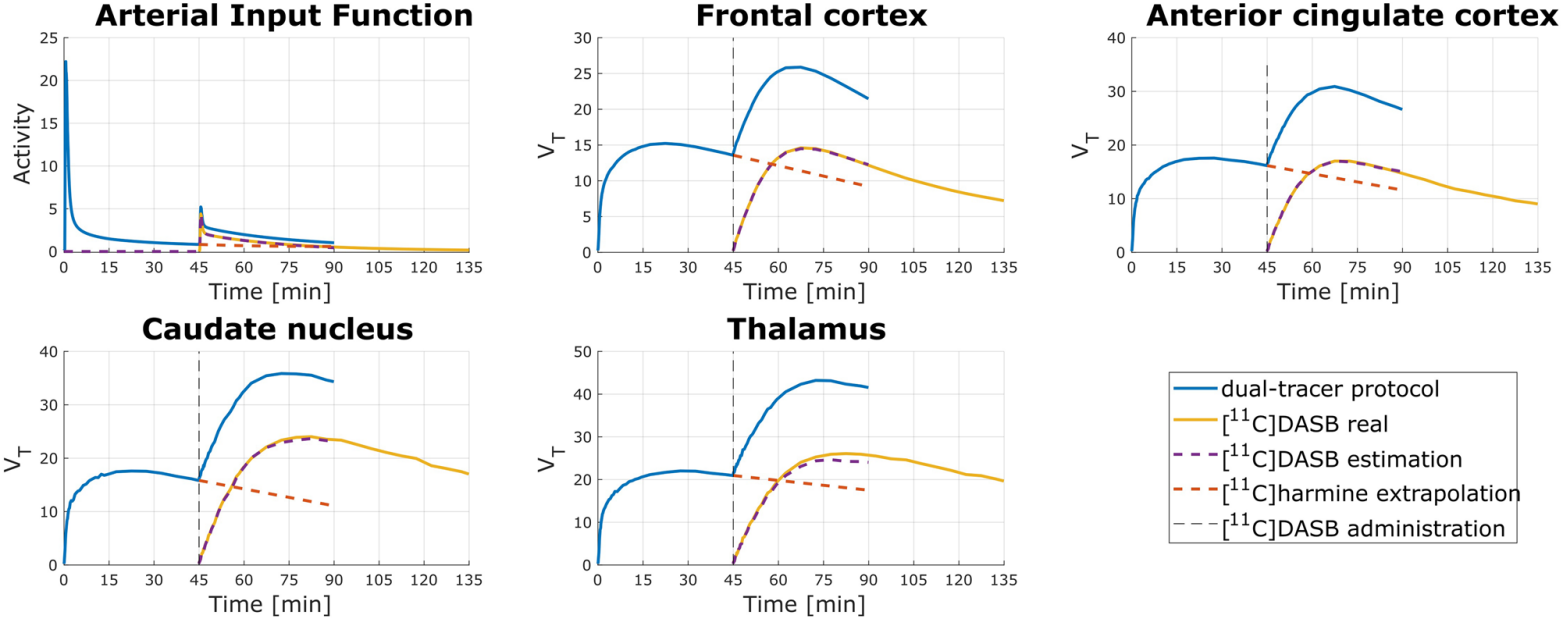
Example TACs and AIFs (top left) when [^11^C]harmine was applied as first. Blue lines TAC_HAR_DASB_ and AIF_HAR_DASB_ are representing dual-tracer application, yellow lines are showing TAC_DASB_ and AIF_DASB_ of 90 min [^11^C]DASB measurement, purple dashed lines shows a time course of simulated TAC_DASB_est_ and AIF_DASB_est_ and the red dashed line express extrapolated TAC_HAR_ext_ and AIF_HAR_ext_. A black vertical dashed line denotes the administration of [^11^C]DASB.

Dual-tracer TACs and AIFs were created for each pair of subjects to simulate the administration of two tracers within one measurement. Examples of both simulation protocols are in Fig. 3 ([^11^C]DASB administered first) and Fig. 4 ([^11^C]harmine administered first) shown as a blue line.

Following the separation of TACs and AIFs, as described in the methods section, V_T_ were calculated for estimated measurement (V_T_DASB_est_, V_T_HAR_est_) as well as for 90 min (V_T_DASB90_, V_T_HAR90_) and 45 min single-tracer measurements (Suppl. Table 2). Afterwards, absolute percentage difference (APD [%]) for each V_T_ comparison was calculated using equations defined in methods section (Eq. 5-8). APD results for the different brain regions are depicted in Table 2.

**Table 2:** Median and interquartile range (IQR) of APD calculated in 13 selected brain regions for the comparison of V_T_DASB_est_ and V_T_DASB45_; V_T_HAR_est_ and V_T_HAR45_; V_T_DASB90_ vs V_T_DASB45_; V_T_HAR90_ and V_T_HAR45_.

ROI	APD_DASB_est_		APD_HAR_est_		APD_HAR45_		APD_DASB45_	
	Median	IQR	Median	IQR	Median	IQR	Median	IQR
THA	6.76	7.35	27.76	39.99	4.31	7.24	16.93	12.78
FRO	4.56	10.38	11.90	35.36	2.23	8.84	15.00	11.40
OCC	10.02	9.45	7.28	36.01	3.90	8.88	12.17	11.42
PAR	5.31	5.63	11.81	36.85	2.54	9.19	14.51	11.45
TMP	12.36	13.57	15.08	27.97	5.63	7.82	14.06	12.67
ACC	2.64	10.88	12.51	34.40	4.87	6.95	15.32	9.11
INS	6.35	5.70	20.00	36.00	3.39	7.97	16.45	12.21
HIP	16.76	11.68	22.29	27.33	6.33	7.79	21.81	8.62
OLF	10.98	34.60	15.42	26.43	6.96	7.33	14.92	9.18
CAU	17.18	15.42	8.35	47.42	4.39	5.39	12.31	11.53
PUT	13.66	14.22	26.83	50.89	2.54	7.20	12.98	8.56
STR	18.86	14.79	26.22	49.17	3.39	5.15	13.84	7.79
CRB	6.76	7.35	21.84	43.87	2.76	1.39	10.42	7.23

**Figure 5:**
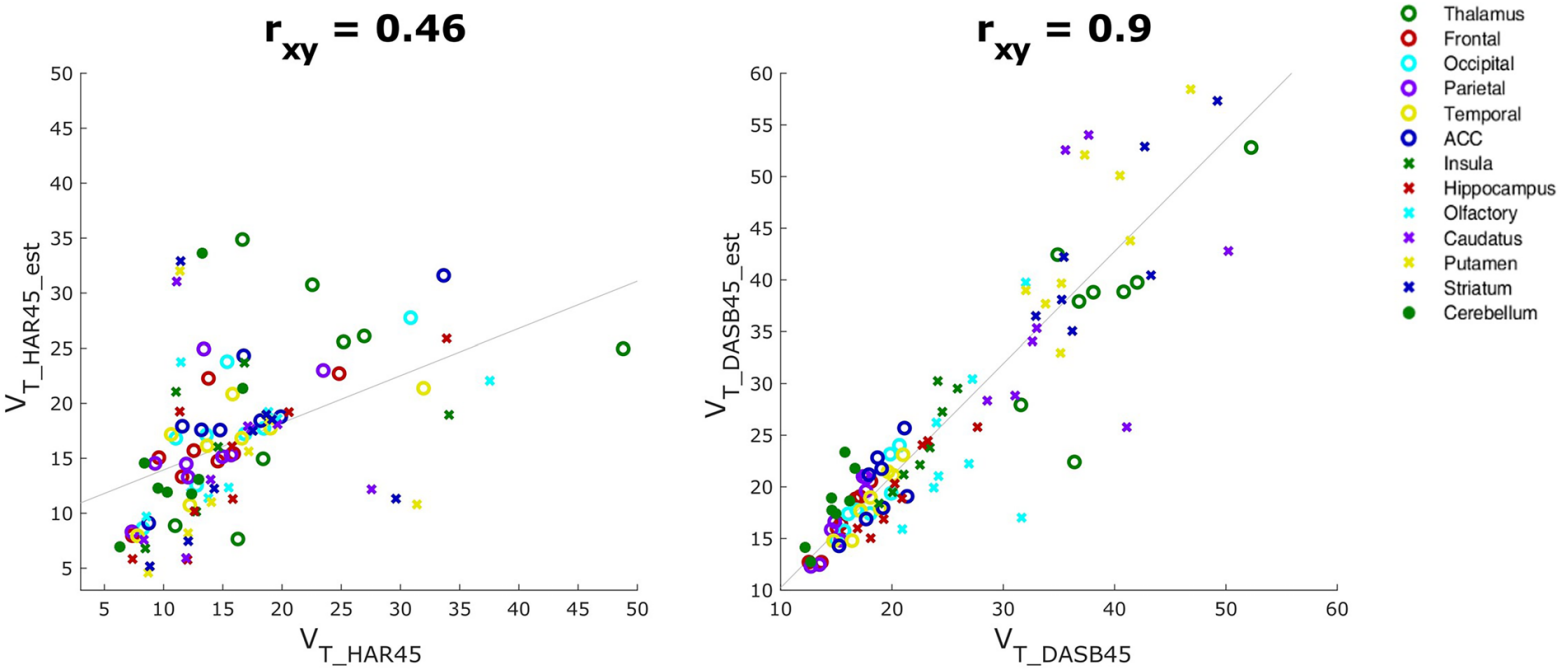
Scatter plots showing the correlation between real and estimated V_T_ of the second tracer V_T_, when [^11^C]DASB is applied first (left) and when [^11^C]harmine is applied first (right). All 13 ROIs considered for the analysis are shown in the figure label with different makes in different colours.

The relation between V_T_ based on real and estimated data, i.e. V_T_HAR_est_ vs V_T_HAR45_ and V_T_DASB_est_ vs V_T_DASB45_ respectively, are shown in Fig. 5. When applying [^11^C]DASB first (Fig. 5, left), V_T_ in ROIs for [^11^C]harmine is underestimated, resulting in a correlation coefficient of r_xy_ = 0.46. In contrast, injection of [^11^C]harmine first is resulting in rather similar V_T_ (r_xy_ = 0.90) in different ROIs for [^11^C]DASB when comparing real and estimated values (Fig. 5, right).

Follow up, comparison between the original scan protocol and 45 min long measurement unveiled a high correlation for both tracers used for simulation. Comparing V_T_DASB90_ to V_T_DASB45_ inferred r_xy_ = 0.95 (Suppl. Fig. 1, left), while comparison of V_T_HAR90_ to V_T_HAR45_ showed even higher correlation of r_xy_ = 0.98 (Suppl. Fig. 1 right).

## Discussion

### ‘Two-in-one-pot’-synthesis

Exemplarily, this simultaneous production was successfully performed for the two commonly used brain PET-tracers, [^11^C]harmine and [^11^C]DASB. For the first time, two carbon-11 tracers were produced in a one-pot synthesis, without the need of splitting the [^11^C]CH_3_I upfront or the need of a second HPLC System. The synthesis in combination with the self-constructed module part was also successfully tested on the new GE TRACERlab™ FX2 C.

Any ^11^C-methylation using [^11^C]CH_3_I occurs via a nucleophilic substitution (SN2 reaction) with iodide as leaving group. For the here described intermolecular reaction, two nucleophiles, a secondary alkyl amine for DASB and a phenolic hydroxide for harmine, compete for the sub-stoichiometric amount of [^11^C]CH_3_I. As all other reaction parameters are similar, the preferred product is only defined by the nucleophilicity of the reactant and kinetic considerations. The formation of both products in the same ratio was surprising as, in general, amines should be preferred over phenols considering their nucleophilicity. However, this equal substrate specificity could only be achieved by deprotonation of the phenolic hydroxy group with strong bases, such as NaOH. Also, other ‘two-in-one-pot’ reactions could be possible as long as the used tracers show similar reaction parameters.

**Figure 6:**
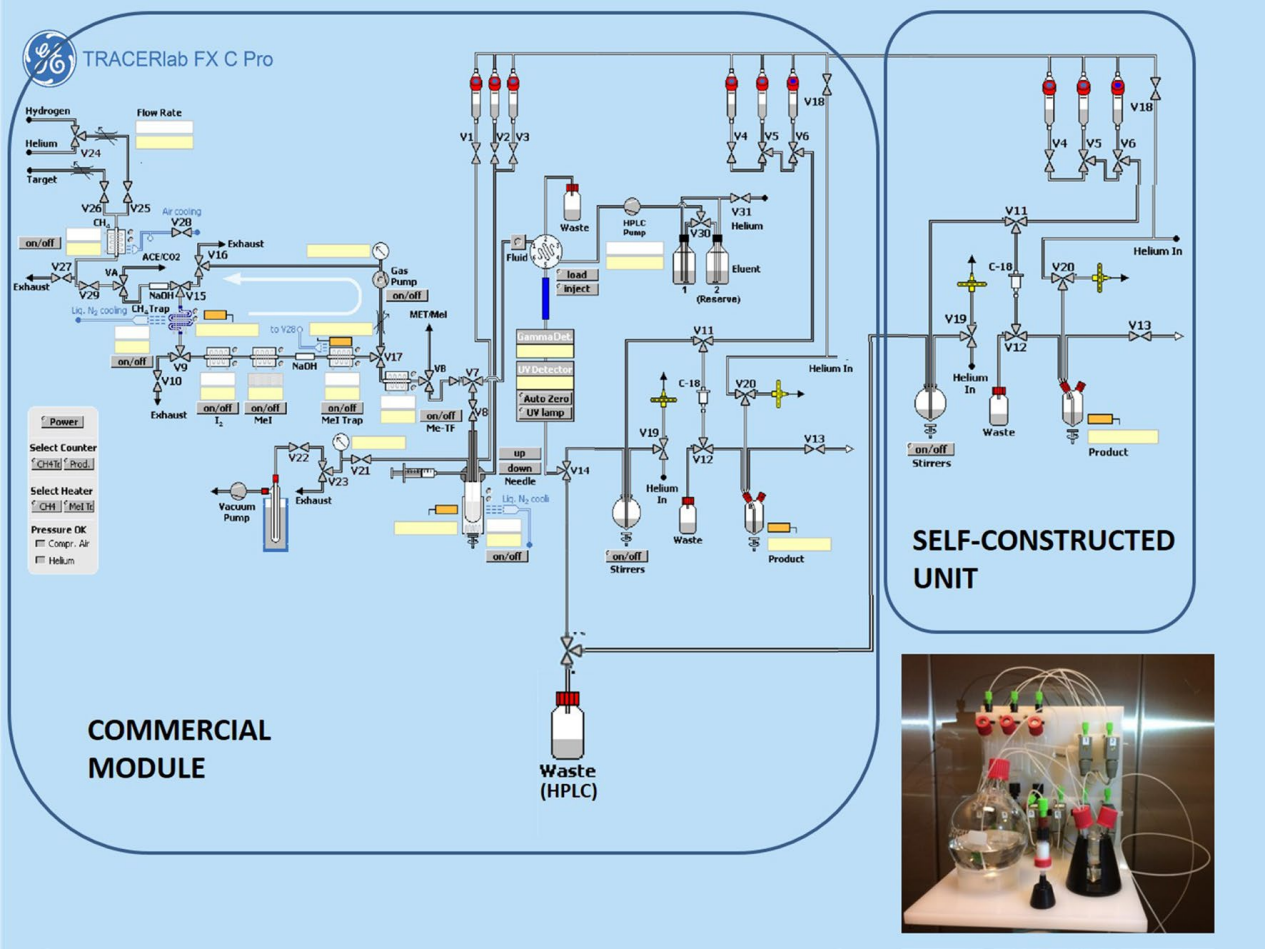
Illustration of the DUAL-synthesis module. The first part contains the commercially available GE Tracerlab FX C Pro synthesizer, on which the first eluted product is further purified from the mobile phase and formulated for application. The second part includes the self-constructed unit, on which the processing of the second (eluted) product takes place (photo below). This part is controlled semi-automated. Sterile filtration is performed in a separate laminar air flow hot cell (not illustrated in this scheme).

The two tracers can be processed together either for a parallel application in two patients (two PET scanners required for dynamic protocols) or time-shifted application in one patient. Both products can be collected in the same bulb and further processed through the original system (Fig. 6, commercial module). In general, this simultaneous radiopharmaceutical preparation led to a significant reduction of radiation burden (− 50%), operator time (− 50%), and overall costs (− 46.2%) in comparison to two single syntheses (100%).

### Quality control

The adaption of the published QC RP-HPLC methods of the respective tracers to the QC DUAL involves an extension of the run time to 5 min, which is still 1 min shorter than using the two single methods consecutively. Furthermore, all potential radiochemical impurities and cross-contaminations of the different products can be detected with this slightly modified RP-HPLC assay. No cross-contamination of the radiotracers or other radiochemical impurities higher than 3% were detected, which meets European regulations for the application to patients. In conclusion, the quality of both radiopharmaceuticals produced via the DUAL method complied with the European Pharmacopeia (Eu. Phr.) criteria and were assessable in a single quality control method separating both products^22^.

### Application to patients and controls

According to GMP regulations (Guidelines on Good Manufacturing Practices for radiopharmaceutical products)^23^ one-pot synthesis production of two tracers with the same or different radiolabels is not directly prohibited. However, it is stated that ‘cross-contamination’ should be prevented by the adoption of feasible measures. Among other points it is stated: ‘avoiding the manufacture of different products at the same time, unless they are effectively segregated’. Consequently, the evidence for full separation must be proven for one-pot synthesis to comply with GMP standards.

On European levels (EU Regulation for clinical trials; No. 536/2014) no specific requirements for the manufacture of diagnostic investigational radiopharmaceuticals for direct use in nuclear medicine facilities within clinical trials are stated. Explicitly, the GMP-compliant production is no longer required for diagnostic radiopharmaceuticals even in the case of investigational medicinal products (IMPs) according to EudraLex 4, but can still be imposed by local (national) regulations^24–26^. However, in the operational rules for pharmaceutical productions exacting the Austrian law on drugs [Arzneimittelgesetz (AMG)], radiopharmaceutical productions in nuclear medicine facilities are explicitly exempted in general. Therefore, the necessity of complying with GMP regulations is also not valid in this situation, leaving full responsibility for the whole process directly with the nuclear medicine departments. Additionally, the Austrian national health authority [Agentur für Gesundheit und Ernaehrungssicherheit (AGES)] requests GMP certified precursor materials and toxicity studies of the reference compound as well as standardized quality criteria and the evidence that no cross-contamination can occur.

Summarizing, the concept of a one-pot simultaneously production of two radiotracers for clinical studies is feasible and, according to regulations (at least in Austria), permitted if complete separation is guaranteed.

### Arterial input function and metabolite correction for dual-tracer application

Separation of interfering radiometabolites and the parent compound is necessary to calculate amount of non-metabolized tracer in plasma as a function of time, i.e. the arterial input function. The main challenge in such a multi-tracer application is the radio-HPLC separation of the two parent compounds and their radio-metabolites with respect to the carbon-11 half-life of 20 min. Furthermore, separation of two tracers, with similar physico-chemical characteristics (molecular weight, logP etc.), and from several radiometabolites and in one assay is an analytical challenge, too.

Therefore, the simplest possibility to correct for radiometabolites are mathematically or population-based models. For the described tracers, [^11^C]harmine and [^11^C]DASB, metabolite data of single-tracer measurements are available. Dual-tracer application using a mathematically model should therefore include comparable study populations^27–29^. Notably, the ability of potential radiometabolites to cross the blood brain barrier is low since all described derivatives are more hydrophilic than the parent compounds^30–32^. Therefore, metabolite identification is unnecessary for kinetic modelling and only the amount of parent compound has to be taken into account. Based on the separation of DASB and harmine, which was shown in the analytical and semi-preparative chromatograms, we suppose that a radio-HPLC assay or a cartridge and radio-HPLC combination can be established for quantification and separation of the parent compounds from each other and their radio-metabolites.

### Dual-tracer simulation

Considering that [^11^C]harmine and [^11^C]DASB are important PET brain tracers for imaging the serotonergic system, the multi-tracer concept has potential to overcome day-to-day variations and increase PET-scanner efficiency. Multi-tracer applications may drive more complex study designs in psychiatric research cause to higher practicability performing longitudinal studies and studies including intervention while studying two protein targets simultaneously.

Although the target organ of interest is the same and both tracers are labelled with the same radionuclide, we hypothesized that they can be distinguished based on their pharmacokinetics. In previously published studies, Koeppe et al.^7^ has shown that PET quantification of dual-tracer application of two carbon-11 brain tracers with an injection time delay between 10–20 min are as accurate as conventional single-tracer applications. However, these authors and other groups suggested that not every tracer pair can be applied in one dynamic scan. Hence, general models for the analysis of multi- tracer applications are lacking as accurate results are depending on the tracer’s different pharmacokinetics as binding behaviour (reversible or irreversible) and retention mechanisms (binding, trapping, perfusion etc.)^2^. For simulation of a dual-tracer injection of [^11^C]harmine and [^11^C]DASB, PET data corrected for radiometabolites of single injections in the same healthy controls were used. V_T_ values were calculated for several possible dual-tracer protocols, altering which tracer was applied first and with staggered time of injection (no time-delay, 15, 30, and 45 min).

Shorter intervals between the injections than 45 min did not result in sufficient TAC separation of both kinetics or could not be calculated for every of the 13 brain regions, therefore no results are available for these protocols. When [^11^C]harmine is applied first, V_T_ for investigate ROIs for [^11^C]DASB are similar to V_T_ calculated after single-tracer scan (r_xy_ = 0.90; Fig. 5, right). Moreover, 45 min of [^11^C]harmine measurement is sufficient to replicate V_T_ obtained after 90 min (r_xy_ = 0.98; Suppl. Fig. 1, right). Accuracy of the dual-tracer measurement is supported also by low APD values (see APD_HAR45_ and APD_DASB_est_ in Table 2). Altered order of tracer application did not lead to similar results, particularly, MAO-A distribution was calculated substantially different than in single-tracer measurement (r_xy_ = 0.46; Fig. 5, left), suggesting that initial application of the tracer with faster kinetics ([^11^C]harmine), allows for accurate estimation of second tracer parameters^2,6^. Correction of radiometabolites during the first 45 min TACs of [^11^C]harmine can be acquired without potential interference of [^11^C]DASB and DASB-radiometabolites. Hence, arterial blood sampling and experimental quantification for each of the parent compounds would be possible.

Considering the half-life of ~ 20 min for carbon-11 and a potential impact on the results caused by decreased molar activity, the second injection should not be applied more than 45 min later. Starting with the assumption of a molar activity of 100 GBq/µmol for the tracer applied last ([^11^C]DASB), a 45 min delay in application results in a reduction to 21 GBq/µmol (2.25 half-life) at injection time. This hits exactly the lower application limit (20 GBq/µmol)^33^. Therefore, molar activity is no limitation of dual-tracer application. Taken into account mean yields of 2 GBq (n = 3) for intravenous bolus injection (4.51 ± 0.15 MBq/kg body), after 45 min the remaining yield would range between 351 and 494 MBq. Considering a 75 kg healthy volunteer, application dose would be 338 MBq. To this end, neither molar activity nor activity yields are limiting factors for a dual-tracer approach of [^11^C]harmine and [^11^C]DASB.

To this end, the basis for all kind of combinations is the detailed knowledge of the radiotracer pharmacokinetics including (radio)metabolism. When applying two tracers within one session, different molecular behaviors need to be kept in mind. If the models describing the kinetics of involved radioligands in inspected regions of interest are far from linearity, the separation of the measured PET signal or arterial input function of individual tracers might become ambiguous. We proofed the importance of time-shifted injections in our simulation, where the order of administration affects the quality of the separation of the measured signal. Additional issues might occur due to tracer selectivity to a set of targets. Precisely, similar affinities to the same binding site might lead to masking of the measured signal. Additionally, for clinical application of two tracers potential radiometabolite formation has to be considered and their analysis has to be established.

## Conclusion

We herewith describe the first simultaneous production of two ^11^C-labelled PET-tracers in a ‘two-in-one-pot’ reaction. Both products were in full accordance with quality control parameters fulfilling the standards for parenteral human application and unexpectedly equivalent yields for both reactions were achieved. This simultaneous radiopharmaceutical preparation resulted in a considerable gain in overall efficiency of the production process, supporting significant reduction of radiation burden, amount of operator time and cost. Besides, this method promises significant improvements for clinical studies. For the future, a radiometabolite assay to quantify the unchanged parent compounds, [^11^C]harmine and [^11^C]DASB, will be establish for considering a study with dual-tracer application, which was shown in a simulated kinetic model to be sufficient and as accurate as compared to single-tracer injection when applying [^11^C]harmine first and [^11^C]DASB 45 min later. Syntheses results (yield and molar activity) of [^11^C]DASB are sufficient for tracer injection after 45 min and therefore a dual-tracer study. To this end, our results are highly promising for further studies in view of optimizations of radiosyntheses, hot cell and scanner efficiency as well as multi-tracer imaging for longitudinal and interventional study designs in neuropsychiatric research.

## Materials and methods

### Radiotracer production

Separate preparations of [^11^C]harmine and [^11^C]DASB were performed in-house by the Division of Nuclear Medicine, Medical University of Vienna (MUW) and General Hospital of Vienna, AKH, according to previously published synthesis protocols^16,17^. The comparison of the production costs for the dual-tracer synthesis was calculated based on the cost for two separate syntheses (100%) including expenditures for radionuclide production, personnel and all consumables for radiosynthesis.

### ‘Two-in-one-pot’ radiotracer production

Dual-tracer production runs were performed using a commercially available GE Tracerlab FX C Pro synthesis module (General Electric Medical System, Uppsala, Sweden). 1 mg of the respective precursors, MASB (N-methyl-2-(2-amino-4-cyanophenylthio)-benzylamine) and harmol (7-hydroxy-1-methyl-9H-pyrido[3,4-b]indole) (both obtained from ABX, Radeberg, Germany), were dissolved in DMSO and 1 µL of 5 M NaOH was added to the solution. [^11^C]CH_3_I was subsequently bubbled through the precursor solution. After a reaction time of 2 min at 100 °C, the crude dual-tracer reaction mixture was purified by means of semi-preparative high performance liquid chromatography (HPLC). The synthesis module was expanded with a self-constructed semi-automated formulation unit to ensure parallel solid phase extraction (SPE)-purification and formulation of both tracers after HPLC (Fig. 2).

In detail, [^11^C]CO_2_ was produced within a GE PETtrace 860 cyclotron (General Electric Medical System, Uppsala, Sweden) by a ^14^N(p,α)^11^C nuclear reaction by means of irradiation of a gas target filled with N_2_ (+ 1% O_2_) (Air Liquide Austria GmbH, Schwechat, Austria). The conversion of [^11^C]CO_2_ to [^11^C]CH_3_I was performed in the GE Tracerlab FX C Pro synthesizer by the gas phase method slightly adjusted from Larsen et al.^34^. The cyclotron production of [^11^C]CO_2_ was terminated at desired target activities between 79–100 GBq using a current of 65 µA for 20–30 min. The produced [^11^C]CH_3_I was trapped on-line on a Porapak® N column and finally released by heating the trap to 190 °C directly in a glass reactor containing 1 mg MASB and 1 mg harmol in 500 µL DMSO and 1 µL of 5 M NaOH (one-pot reaction). Subsequently, the reactor was heated to 100 °C for 2 min. After the reaction time, the reactor was cooled to below 35 °C and the reaction was quenched by adding 1 mL of reversed phase (RP)-HPLC solvent. The entire volume of the crude mixture was automatically transferred to a semi-preparative RP-HPLC column. Details of the single preparation methods are described elsewhere^16,17^. From this step on, the product, [^11^C]harmine (see Suppl. Fig. 2, semi-preparative chromatogram) which elute first, was processed on the commercial available module, while [^11^C]DASB was further processed on the self-constructed unit. The [^11^C]harmine peak was collected in a round-bottom flask containing 60 mL of distilled water to dilute the amount of organic solvent in the mobile phase and further purified via SPE (C18plus SepPak®, Waters). After rinsing of the SepPak with water (V6) for complete removal of residual RP-HPLC solvents, the pure product was eluted with 1.5 mL EtOH (V5) into a two-neck vial and the cartridge and transfer lines were rinsed with further 5 mL 0.9% saline into the same vial (commercial module, Fig. 6). After formulation with 9 mL 0.9% saline, 1 mL 3% saline and 1 mL 125 mM phosphate buffer, sterile filtration (0.22 μm) was performed under aseptic conditions (laminar air flow hot cell, class A) to avoid microbial and particular contamination. During the purification and formulation of [^11^C]harmine, baseline separated [^11^C]DASB (elute 2 min apart) was collected in the round-bottom flask, containing 80 mL water, of the self-constructed unit and further purified and formulated as described above (self-constructed unit, Fig. 6).

### Quality control

RP-HPLC analyses were performed on an Agilent 1260 system (Agilent Technologies GmbH; Santa Clara, CA, USA) equipped with a quaternary pump (G1311B), a multi wavelength UV-detector (G1365D), a manual injector (G1328C), a Nal scintillation detector (Gabi *) from Raytest/Elysia and GINA Star 5.9 controlling software (Elysia-Raytest; Straubenhardt, Germany). As stationary phase a X-Bridge BEH Shield RP-18, 4.6 × 50 mm, 2.5 μm, 130 Å (Waters Corp., Milford, MA, USA) was used and the mobile phase consists of D: 50 mM ammonium phosphate, C: water and B: acetonitrile (90%) in different isocratic mixtures or in a gradient method, respectively (Suppl. Table 2). An exemplary analytic chromatogram is illustrated in Suppl. Fig. 3, showing a mixture of the reference standards (harmol, harmine, MASB and DASB) using the QC-DUAL method.

## PET data

The scans were performed in previously reported studies with GE Advance PET scanner at the Division of Nuclear Medicine and in cooperation with the Division of General Psychiatry of the Medical University of Vienna (MUW) and the University Hospital of Vienna (AKH)^18,35^. Hence, the data of these previously conducted PET scans was used retrospectively for the simulation of dual-tracer application. In the studies of Spies et al.^18^ and Gryglewski et al.^35^, PET scans were acquired in 3D mode in a total scanning time of 90 min. For attenuation correction, an additional 5 min transmission scan was performed prior to tracer administration using ^68^Ge rod sources. Additional anatomical T1-weighted magnetic resonance imaging (MRI) scans were acquired with 3 T PRISMA MR Scanner (Siemens Medical, Erlangen, Germany, 1 × 1 mm voxel size, 1.1 mm slice thickness, 200 slices). To simulate the dual-tracer application, we made use of 16 single PET measurements acquired as part of previously published studies. In particular, we adopted eight [^11^C]DASB and 8 [^11^C]harmine PET scans of healthy subjects that are matched by weight, sex and age (Suppl. Table 3). The radiotracers [^11^C]DASB and [^11^C]harmine were intravenously administered as bolus with 4.51 ± 0.15 MBq/kg body weight and 4.66 ± 0.19 MBq/kg body weight, respectively.

All participants provided written informed consent and received financial reimbursement for participation. This study was approved by the Ethics Committee of the Medical University of Vienna and performed according to the Declaration of Helsinki.

## Arterial blood sampling and analysis of metabolites in plasma

Arterial blood samples during [^11^C]harmine scan were taken automatically for the first 10 min complemented with manual samples at 1, 5, 10, 20, 30, 45, 60, and 80 min after bolus application, while automatic blood sampling lasted 3 min and was accompanied with manual samples taken at 1, 5, 10, 20, 40, and 70 min after injection of [^11^C]DASB. Arterial input function (AIF) was obtained as a product of multiplication of plasma-to-whole blood ratio, whole blood activity and the fraction of intact radioligand in the plasma are according to the respective published protocols^31,32^.

## Data processing

Dynamic PET scans were corrected for motion, co-registered to individual T1-weighted structural MR images and normalized to MNI space using SPM8 (Wellcome Trust Centre for Neuroimaging, London, United Kingdom). Subsequently, time activity curves (TACs) were extracted for the regions of interest (ROIs) previously associated with either MAO-A enzyme distribution^18^ or SERT density analysis^28^ including frontal (FRO), temporal (TMP), parietal (PAR) and occipital (OCC) cortex, anterior cingulate cortex (ACC), insula (INS), hippocampus (HIP), caudate nucleus (CAU), putamen (PUT), thalamus (THA), striatum (STR) and cerebellar grey matter (CRB) were extracted using Automated Anatomical Labelling (AAL) atlas^36^.

## Dual-tracer measurements simulation

For multi-tracer simulation, AIFs and TACs were resampled so that the time difference between the two samples was 5 s. Simulation of a dual-tracer application was examined using [^11^C]DASB (TAC_DASB_, AIF_DASB_) and [^11^C]harmine (TAC_HAR_, AIF_HAR_) bolus data, where one tracer is administered simultaneously with PET start and the second 15, 30, and 45 min later. The simulation was done by summation of the single-tracer measurements, where data points of the second tracer are shifted by 15, 30, or 45 min with the whole simulation lasting 90 min. Both possible orders of administration were examined, i.e. [^11^C]DASB first (TAC_DASB_HAR_, AIF_DASB_HAR_) and [^11^C]harmine first (TAC_HAR_DASB_, AIF_HAR_DASB_), respectively. Based on the first 15, 30, or 45 min of dual-tracer simulation, TACs and AIFs of the first tracer were extrapolated (TAC_DASB_ext_, AIF_DASB_ext_; TAC_HAR_ext_, AIF_HAR_ext_) to the measurement duration of 90 min. Subsequently, TACs and AIFs of the second tracer (TAC_DASB_est_, AIF_DASB_est_; TAC_HAR_est_, AIF_HAR_est_) were calculated as follows:

1 $$ {\text{TAC}}_{{{\text{HAR\_est}}}} = {\text{TAC}}_{{{\text{DASB\_HAR}}}} - {\text{TAC}}_{{{\text{DASB\_ext}}}} $$

2 $$ {\text{TAC}}_{{{\text{DASB\_est}}}} = {\text{TAC}}_{{{\text{HAR\_DASB}}}} - {\text{TAC}}_{{{\text{HAR\_ext}}}} $$

3 $$ {\text{AIF}}_{{{\text{HAR\_est}}}} = {\text{AIF}}_{{{\text{DASB\_HAR}}}} - {\text{AIF}}_{{{\text{DASB\_ext}}}} $$

4 $$ {\text{AIF}}_{{{\text{DASB\_est}}}} = {\text{AIF}}_{{{\text{HAR\_DASB}}}} - {\text{AIF}}_{{{\text{HAR\_ext}}}} $$

Following the previous use of graphical Logan’s plot with metabolite corrected AIF for MAO-A^32^ and DASB^35^, the total volume of distribution (V_T_) in 13 ROIs for both tracers was quantified (V_T_DASB90_; V_T_HAR90_). As dual-tracer TACs and AIFs for the simulation with the time difference of 15 and 30 min between applications were not possible to separate to single-tracer measurements by the extrapolation, we used only protocols with 45 min between tracer administration. In addition, V_T_ were calculated for simulated measurements (V_T_HAR_est_; V_T_DASB_est_) and for the first 45 min of dual-tracer measurement (V_T_DASB45_; V_T_HAR45_), which is equal to the first 45 min of the single-tracer measurement.

Using Pearson's correlation, real and estimated V_T_ were compared (V_T_HAR_est_ vs V_T_HAR45_; V_T_DASB_est_ vs V_T_DASB45_) across all ROIs and subjects. Additionally, the relation between V_T_HAR90_ vs V_T_HAR45_ and V_T_DASB90_ vs V_T_DASB45_ was investigated to verify whether the potential 45 min protocol would bring similar results as the original scanning protocol. In addition, absolute percentage difference (APD [%]) in ROIs was calculated for all aforementioned comparisons and subsequently averaged over subjects.

5 $$ {\text{APD}}_{{{\text{HAR\_est}}}} = \left| {{\text{V}}_{{{\text{T\_HAR\_est}}}} - {\text{V}}_{{{\text{T\_HAR45}}}} } \right|/{\text{V}}_{{{\text{T\_HAR45}}}} \cdot 100 $$

6 $$ {\text{APD}}_{{{\text{DASB\_est}}}} = \left| {{\text{V}}_{{{\text{T\_DASB\_est}}}} - {\text{V}}_{{{\text{T\_DASB}}45}} } \right|/{\text{V}}_{{{\text{T\_DASB}}45}} \cdot 100 $$

7 $$ {\text{APD}}_{{{\text{HAR}}45}} = \left| {{\text{V}}_{{{\text{T\_HAR}}45}} - {\text{V}}_{{{\text{T\_HAR}}90}} } \right|/{\text{V}}_{{{\text{T\_HAR}}90}} \cdot 100 $$

8 $$ {\text{APD}}_{{{\text{DASB}}45}} = \left| {{\text{V}}_{{{\text{T\_DASB}}45}} - {\text{V}}_{{{\text{T\_DASB}}90}} } \right|/{\text{V}}_{{{\text{T\_DASB}}90}} \cdot 100 $$

## Supplementary Information

Supplementary Information.
