# Supplementary material for: Simultaneous radiomethylation of [11C]harmine and [11C]DASB and kinetic modeling approach for serotonergic brain imaging in the same individual
Source: Sci Rep. 2022 Feb 28;12:3283. doi: 10.1038/s41598-022-06906-0 (PMC8885643; doi:10.1038/s41598-022-06906-0)
Supplement: Supplementary file 1 — Supplementary Information. [file 41598_2022_6906_MOESM1_ESM.docx]

**Supplementary information**

**Simultaneous radiomethylation of [^11^C]harmine and [^11^C]DASB and kinetic modeling approach for serotonergic brain imaging in the same individual**

## Chrysoula Vraka^1^, Matej Murgaš^2^, Lucas Rischka^2^, Barbara Katharina Geist^1^, Rupert Lanzenberger^2^, Gregor Gryglewski^2^, Thomas Zenz^1^, Wolfgang Wadsak^1,3^, Markus Mitterhauser^1,4^, Marcus Hacker^1^, Cécile Philippe^1^*, Verena Pichler^5^

^1^ Department of Biomedical Imaging and Image-guided Therapy, Medical University of Vienna, Vienna, Austria

^2^ Department of Psychiatry and Psychotherapy, Medical University of Vienna, Vienna, Austria

^3^ CBmed GmbH, Center for Biomarker Research in Medicine, Graz, Austria

^4^ Ludwig Boltzmann Institute Applied Diagnostics, Vienna, Austria

^5^ Department of Pharmaceutical Sciences, Division of Pharmaceutical Chemistry, University of Vienna, Vienna, Austria

^*^Correspondence: cecile.philippe@meduniwien.ac.at; Tel: +43-1-40400-20340

| HPLC Method | Mobile Phase | | | |
| --- | --- | --- | --- | --- |
| QC_Harmine:  1 mL/min flowrate  246 nm  3 min run time:  Rt harmol: 0.39 min;  Rt harmine 1.35 min | B: 35%  C: 39%  D: 26% | | | |
| QC_DASB:  1 mL/min flowrate  254 nm  3 min run time:  Rt MASB 0.21 min; Rt DASB 0.54 min | B: 68%  C: 16%  D: 16% | | | |
| QC_DUAL  1 mL/min flowrate  246 & 254 nm  5 min run time:  Rt harmol: 0.39 min;  Rt harmine 1.35 min  Rt MASB 2.14 min; Rt DASB 3.36 min | **Time** | **%B** | **%C** | **%D** |
|  | 0-1.50 | 35 | 39 | 26 |
|  | 1.50-2.30 | 80 | 4 | 16 |
|  | 2.30-4.00 | 80 | 4 | 16 |
|  | 4.00-4.30 | 35 | 39 | 26 |
|  | 4.30-5.00 | 35 | 39 | 26 |

**Suppl. Table 1.** Overview on the used mobile phases for each HPLC assay applied for quality control (QC). B: acetonitrile/water (90/10 %v/v); C: water; D: 50 mM ammonium phosphate (pH 9.3).

| ROI | V_T_DASB45_ | V_T_DASB_est_ | V_T_DASB90_ | V_T_HAR45_ | V_T_HAR_est_ | V_T_HAR90_ |
| --- | --- | --- | --- | --- | --- | --- |
|  | Mean ± SD | Mean ± SD | Mean ± SD | Mean ± SD | Mean ± SD | Mean ± SD |
| THA | 39.11 ± 6.24 | 37.62 ± 9.16 | 47.42 ± 8.57 | 23.23 ± 11.57 | 21.72 ± 10.06 | 22.75 ± 9.60 |
| FRO | 15.51 ± 1.82 | 16.40 ± 2.92 | 18.05 ± 2.34 | 13.77 ± 5.24 | 15.90 ± 4.76 | 13.57± 4.57 |
| OCC | 17.76 ± 2.18 | 18.72 ± 3.31 | 20.12 ± 2.42 | 15.91 ± 6.85 | 17.70 ± 5.96 | 15.32± 5.45 |
| PAR | 15.47 ± 1.93 | 16.70 ± 3.50 | 17.73 ± 1.97 | 13.52 ± 4.89 | 16.13 ± 5.35 | 13.30± 4.29 |
| TMP | 18.27 ± 2.08 | 18.73 ± 3.05 | 21.21 ± 2.59 | 15.97 ± 7.37 | 16.10 ± 4.63 | 15.50± 5.82 |
| ACC | 18.79 ± 1.96 | 19.96 ± 3.63 | 21.90 ± 2.35 | 17.10 ± 7.60 | 19.42 ± 6.43 | 16.97± 6.63 |
| INS | 22.55 ± 2.40 | 24.00 ± 4.53 | 26.47 ± 3.04 | 16.81 ± 7.83 | 16.61 ± 5.59 | 16.49 ± 6.51 |
| HIP | 21.13 ± 3.41 | 20.17 ± 4.16 | 26.54 ± 4.50 | 16.19 ± 8.14 | 14.19 ± 7.13 | 16.50 ± 7.77 |
| OLF | 26.33 ± 3.92 | 24.07 ± 7.90 | 30.42 ± 5.20 | 17.20 ± 9.01 | 16.12 ± 5.41 | 17.09 ± 7.62 |
| CAU | 36.23 ± 6.87 | 37.72 ± 10.96 | 40.31 ± 6.14 | 15.87 ± 6.02 | 15.44 ± 7.84 | 15.87 ± 6.20 |
| PUT | 37.78 ± 4.86 | 44.22 ± 8.59 | 42.95 ± 6.12 | 16.54 ± 7.02 | 14.96 ± 8.50 | 16.71 ± 6.98 |
| STR | 39.17 ± 5.46 | 45.71 ± 10.59 | 44.32 ± 6.26 | 16.45 ± 6.48 | 15.52 ± 8.68 | 16.77 ± 6.84 |
| CRB | 14.71 ± 1.59 | 18.09 ± 3.53 | 16.10 ± 1.70 | 11.23 ± 3.26 | 15.69 ± 8.28 | 10.93 ± 3.37 |

**Suppl. Table 2**: Mean and standard deviation of V_T_ calculated in 13 selected brain regions for real single-tracer measurement with 45 min protocol (V_T_DASB45_; V_T_HAR45_), real single-tracer measurement with 90min protocol (V_T_DASB90_; V_T_HAR90_) and simulated measurement with 45 min protocol when tracer was administered as the second (V_T_HAR_est_; V_T_DASB_est_). Frontal (FRO), temporal (TMP), parietal (PAR) and occipital (OCC) cortex, anterior cingulate cortex (ACC), insula (INS), hippocampus (HIP), caudate nucleus (CAU), putamen (PUT), thalamus (THA), striatum (STR) and cerebellar grey matter (CRB).

| Demographic Data | | Mean ± SD |
| --- | --- | --- |
| DASB | Sex | 3F |
|  |  | 5M |
|  | Weight (kg) | 69.63 ± 9.23 |
|  | Age (years) | 26.25 ± 2.90 |
| Harmine | Sex | 3F |
|  |  | 5M |
|  | Weight (kg) | 70.00 ± 9.85 |
|  | Age (years) | 30.50 ± 10.22 |

**Suppl. Table 3**. Demographic data of subjects included in the simulation analysis including average age and weight difference of matched pairs; F=female, M=male, Mean ± standard deviation.


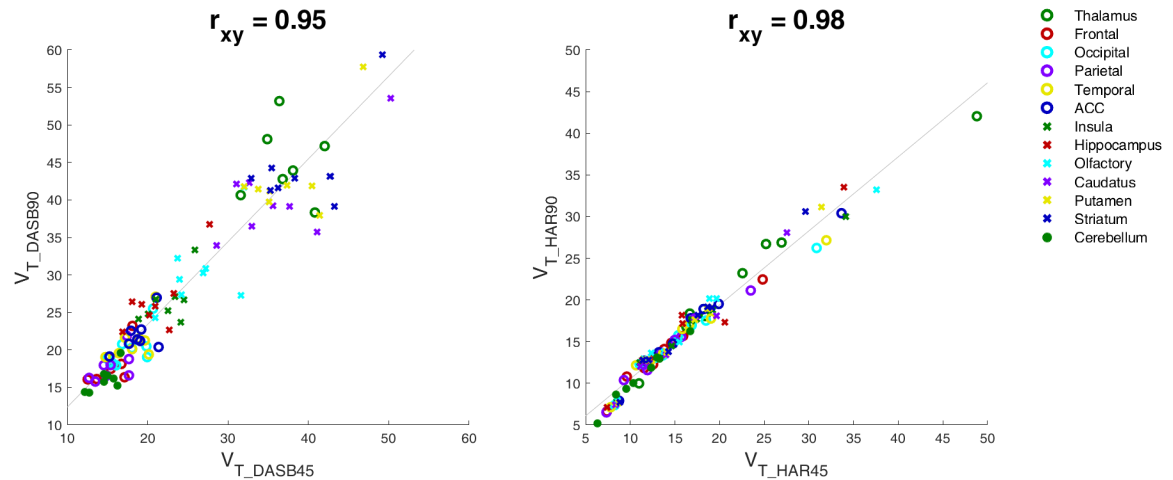


**Suppl. Figure 1.** Scatter plots showing the correlation between VT based on 45 and 90 min long measurements, using [^11^C]DASB (left) and [^11^C]harmine (right). All 13 ROIs considered for the analysis are shown in the figure label with different makes in different colours.


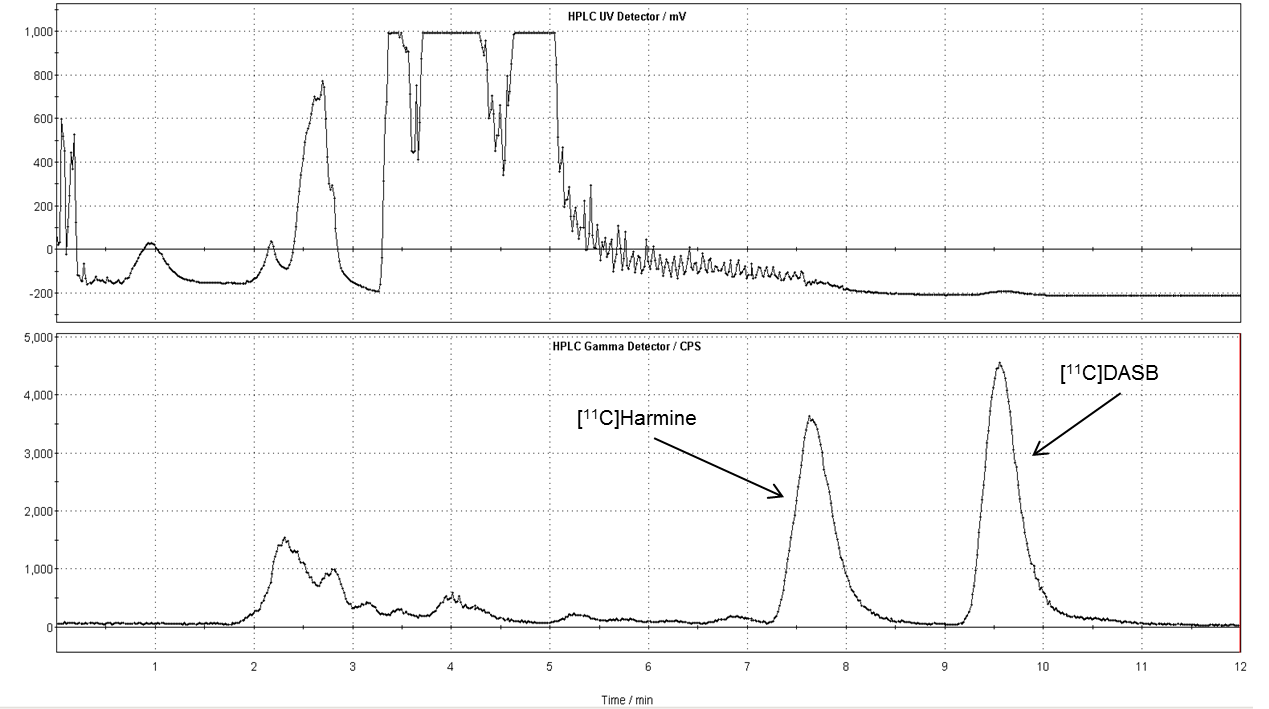


**Suppl. Fig. 2.** Exemplary semi-preparative RP-HPLC chromatogram for the separation of [^11^C]harmine and [^11^C]DASB in a single HPLC run.


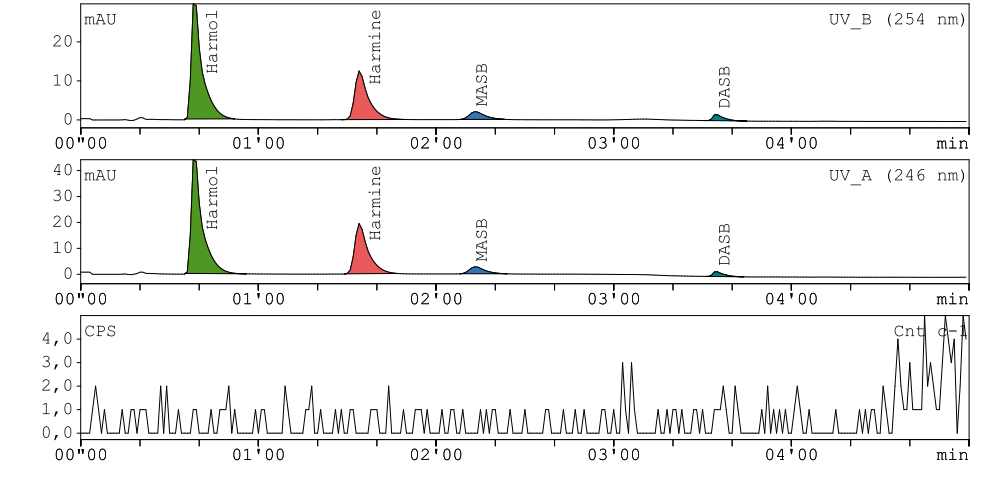


**Suppl. Fig. 3.** Representative analytical chromatogram of the reference standards mixture. The RP-HPLC method for the separation of the products and precursors is described in table 1. Cross-contaminations as well as the product and precursor concentration can be analysed within 4 min.
